# Genome Sequence of the Quorum-Quenching *Rhodococcus erythropolis* Strain R138

**DOI:** 10.1128/genomeA.00224-14

**Published:** 2014-03-27

**Authors:** Anthony Kwasiborski, Samuel Mondy, Amélie Beury-Cirou, Denis Faure

**Affiliations:** a Institut des Sciences du Végétal, CNRS, Gif-sur-Yvette, France; b Comité Nord Plant de Pomme de Terre (CNPPT), Semences, Innovation, Protection Recherche et Environnement (SIPRE), Achicourt, France

## Abstract

*Rhodococcus erythropolis* strain R138 was isolated from the rhizosphere of *Solanum tuberosum* and selected for its capacity to degrade *N*-acyl-homoserine lactones, quorum-sensing signals used as communication molecules by the potato pathogens *Pectobacterium* and *Dickeya*. Here, we report the genome sequence of *Rhodococcus erythropolis* strain R138.

## GENOME ANNOUNCEMENT

*Proteobacteria* may use quorum-sensing (QS) signals, such as *N*-acyl-homoserine lactones (AHLs), to synchronize the gene expression at a population level. Several bacteria, including *Rhodococcus erythropolis*, are able to degrade AHLs, hence disrupting the expression of the QS-regulated functions (1, 2). These AHL-degrading bacteria were collectively named quorum-quenching bacteria (3). *R. erythropolis* expresses at least three enzymatic activities involved in AHL degradation: lactonase, opening the gamma-butyrolactone ring of the AHLs; amidase, cleaving AHLs into homoserine lactone and fatty acids; and reductase, converting the ketone function at the C-3 position of the fatty chain into hydroxyl (4, 5). Until now, only the lactonase-coding gene *qsdA* was characterized (5). The quorum-quenching *Rhodococcus* strains are proposed to be used as antibiofouling (6) and biocontrol (1, 7) agents. *R. erythropolis* strain R138 was isolated from the potato rhizosphere (7). It is able to degrade AHLs and limit the symptoms induced by the plant pathogen *Pectobacterium atrosepticum* on potato tubers (8). The growth and root colonization of *R. erythropolis* R138 are enhanced in the presence of gamma-caprolactone and gamma-heptanolactone, which are assimilated as a carbon source (9). In *R. erythropolis* R138, the lactonase QsdA is involved in the cleavage of AHLs, as well as that of gamma-caprolactone and gamma-heptanolactone (5, 10).

Here, we report the *de novo* genome assembly of *R. erythropolis* R138 by combining Illumina and 454-Roche technologies. Three genomic libraries were constructed: a 300-bp library used for paired-end 2 × 72 Illumina sequencing (Imagif, France), a 380-bp shotgun library used for single-read 454 sequencing, and a long paired-end library with an insert size of 8 kbp used for 454 sequencing (Eurofins MWG, Germany). Sequence reads with low quality (<0.05), ambiguous nucleotides (*n* > 2), and sequence lengths of <50 (454 mate-paired), 20 (454 single), or 70 (Illumina) nucleotides were discarded for the assembly. Assembly was carried out using the CLC Genomics Workbench version 5.1 (CLC bio, Aarhus, Denmark), with a read length of 0.5 and similarity of 0.8 as parameters. In total, 53,576,242 reads were obtained, corresponding to 4,010,660,803 bases, with an average length of 74.9 bp. The scaffolding was processed using SSPACE basic version 2.0 (11). The *in silico* finishing of the remaining gaps was carried out by mapping mate-pair reads (read length, 0.9; similarity, 0.95) on each of the 5-kbp contig ends. Next, the collected reads were used for *de novo* assembly (read length, 0.5; similarity, 0.8). Some additional gaps were resolved using Sanger sequencing of the PCR amplicons. The published sequence is composed of 12 contigs, with a sequence length from 5.5 kbp to 2.7 Mbp grouped in 3 scaffolds.

The *R. erythropolis* R138 genome consists of one circular chromosome (6,444,743 bp), a linear plasmid (247,675 bp), and a circular plasmid (84,151 bp). The G+C content percentages were homogenous among the replicons (from 60 to 62%). A total of 6,562 coding sequences were predicted using the Rapid Annotations using Subsystems Technology (RAST) version 4.0 automated pipeline (12).

### Nucleotide sequence accession number.

This whole-genome shotgun project has been deposited at DDBJ/EMBL/GenBank under the accession no. ASKF00000000.

## References

[1] d’Angelo-Picard C Faure D Penot I Dessaux Y 2005 Diversity of *N*-acyl homoserine lactone-producing and -degrading bacteria in soil and tobacco rhizosphere. Environ. Microbiol. 7:1796–1808. 10.1111/j.1462-2920.2005.00886.x 16232294

[2] Uroz S D’Angelo-Picard C Carlier A Elasri M Sicot C Petit A Oger P Faure D Dessaux Y 2003 Novel bacteria degrading *N*-acylhomoserine lactones and their use as quenchers of quorum-sensing-regulated functions of plant-pathogenic bacteria. Microbiology 149:1981–1989. 10.1099/mic.0.26375-0 12904538

[3] Dong YH Wang LH Xu JL Zhang HB Zhang XF Zhang LH 2001 Quenching quorum-sensing-dependent bacterial infection by an *N*-acyl homoserine lactonase. Nature 411:813–817. 10.1038/35081101 11459062

[4] Uroz S Chhabra SR Cámara M Williams P Oger P Dessaux Y 2005 *N*-acylhomoserine lactone quorum-sensing molecules are modified and degraded by *Rhodococcus erythropolis* W2 by both amidolytic and novel oxidoreductase activities. Microbiology 151:3313–3322. 10.1099/mic.0.27961-0 16207914

[5] Uroz S Oger PM Chapelle E Adeline MT Faure D Dessaux Y 2008 A *Rhodococcus qsdA*-encoded enzyme defines a novel class of large-spectrum quorum-quenching lactonases. Appl. Environ. Microbiol. 74:1357–1366. 10.1128/AEM.02014-07 18192419 PMC2258624

[6] Kim SR Oh HS Jo SJ Yeon KM Lee CH Lim DJ Lee CH Lee JK 2013 Biofouling control with bead-entrapped quorum quenching bacteria in membrane bioreactors: physical and biological effects. Environ. Sci. Technol. 47:836–842. 10.1021/es303995s 23256502

[7] Cirou A Diallo S Kurt C Latour X Faure D 2007 Growth promotion of quorum-quenching bacteria in the rhizosphere of *Solanum tuberosum*. Environ. Microbiol. 9:1511–1522. 10.1111/j.1462-2920.2007.01270.x 17504488

[8] Cirou A Raffoux A Diallo S Latour X Dessaux Y Faure D 2011 Gamma-caprolactone stimulates growth of quorum-quenching *Rhodococcus* populations in a large-scale hydroponic system for culturing *Solanum tuberosum*. Res. Microbiol. 162:945–950. 10.1016/j.resmic.2011.01.010 21288487

[9] Cirou A Mondy S An S Charrier A Sarrazin A Thoison O Dubow M Faure D 2012 Efficient biostimulation of native and introduced quorum-quenching *Rhodococcus erythropolis* populations is revealed by a combination of analytical chemistry, microbiology, and pyrosequencing. Appl. Environ. Microbiol. 78:481–492. 10.1128/AEM.06159-11 22081576 PMC3255727

[10] Barbey C Crépin A Cirou A Budin-Verneuil A Orange N Feuilloley M Faure D Dessaux Y Burini JF Latour X 2012 Catabolic pathway of Gamma-caprolactone in the biocontrol agent *Rhodococcus erythropolis*. J. Proteome Res. 11:206–216. 10.1021/pr200936q 22085026

[11] Boetzer M Henkel CV Jansen HJ Butler D Pirovano W 2011 Scaffolding pre-assembled contigs using SSPACE. Bioinformatics 27:578–579. 10.1093/bioinformatics/btq683 21149342

[12] Aziz RK Bartels D Best AA DeJongh M Disz T Edwards RA Formsma K Gerdes S Glass EM Kubal M Meyer F Olsen GJ Olson R Osterman AL Overbeek RA McNeil LK Paarmann D Paczian T Parrello B Pusch GD Reich C Stevens R Vassieva O Vonstein V Wilke A Zagnitko O 2008 The RAST server: Rapid Annotations using Subsystems Technology. BMC Genomics 9:75. 10.1186/1471-2164-9-75 18261238 PMC2265698

